# Cardiovascular mortality in a Swedish cohort of female industrial workers exposed to noise and shift work

**DOI:** 10.1007/s00420-020-01574-x

**Published:** 2020-09-06

**Authors:** Helena P. Eriksson, Mia Söderberg, Richard L. Neitzel, Kjell Torén, Eva Andersson

**Affiliations:** 1 Department of Occupational and Environmental Medicine, Sahlgrenska University Hospital, Göteborg, Sweden; 2 Occupational and Environmental Medicine, School of Public Health and Community Medicine, Institute of Medicine, Sahlgrenska Academy, University of Gothenburg, BOX 414, 405 30 Göteborg, Sweden; 3 Department of Environmental Health Sciences, University of Michigan, Ann Arbor, MI USA

**Keywords:** Cerebrovascular disease, JEM, Myocardial infarction, Night work, Noise, Paper mills

## Abstract

**Purpose:**

The aim was to study mortality due to cardiovascular disease as well as total mortality, among female industrial workers, and the association to occupational noise and shift work.

**Methods:**

Women from cohorts of soft tissue paper mills (*N* = 3013) and pulp and paper mills (*N* = 1483) were merged into one cohort. Job exposure matrices were developed and used for classification of shift work and noise exposure. Every year was classified as shift work excluding nights or shift work including nights. Noise was classified into seven 5 dB(A) bins from < 75 to ≥ 100 dB(A). Mortality from cardiovascular diseases and total mortality during 1956–2013 was calculated as a standardized mortality ratio (SMR) with 95% confidence interval (CI) using the female general population as a reference.

**Results:**

Fatal myocardial infarctions (*N* = 144) were increased in the total cohort, SMR 1.20 (95% CI 1.01–1.41) but not total mortality. The SMR for myocardial infarction for women exposed to noise ≥ 90 dB(A) for > 10 years was 1.41 (95% CI 1.02–1.89) and for those exposed to night shifts > 10 years, 1.33 (95% CI 0.91–1.89). Shift workers without nights ≤ 65 years, with noise exposure ≥ 90 dB(A), had SMR 2.41 (95% CI 1.20–4.31) from myocardial infarction. There was no increased mortality from cerebrovascular disease.

**Conclusions:**

Female paper mill workers had an increased mortality from acute myocardial infarction, especially before retirement age, when exposed to noise ≥ 90 dB(A) and with long-time employment. Exposure to shift work and noise usually occurred concurrently.

## Background

Shift work is a commonly occurring form of work. In Sweden, one of five employees works on a shift schedule according to an investigation by Statistics Sweden in 2018 (Statistics Sweden 2018). There are different definitions, but shift work is commonly defined as all work that is not scheduled during the daytime, (e.g., outside the hours between 7–8 am and 5–6 pm) (IARC 2010). Shift work can disturb the diurnal rhythm, sleep and social life, which can influence well-being and health (Boivin and Boudreau 2014). Studies have indicated that shift work can increase the risk of coronary heart disease. A systematic review and meta-analysis from 2012 concluded that shift work is associated with increased risk for myocardial infarction with a relative risk of 1.23 (95% confidence interval, CI 1.15–1.31) (Vyas et al. 2012). Most shift work studies on women have investigated nurses (Vetter et al. 2016). There are few studies of industrially employed female shift workers and health risks, especially regarding cardiovascular disease.

Women within the industrial sector are also subjected to other hazardous exposures such as noise. It is well known that noise can cause various health effects, in particular hearing loss (Lie et al. 2016). A 2016 systematic review found a strong association between occupational noise exposure and high blood pressure but scientific evidence was limited for associations with other types of cardiovascular disease (Skogstad et al. 2016). However, a longitudinal cohort study of 5753 men found that occupational noise was associated with an increased risk of coronary heart disease but not of stroke (Eriksson et al. 2018). Exposure to noise and shift work often occurs concurrently in industrial settings such as the paper industry (Virkkunen et al. 2006), but few studies have simultaneously assessed the health risk of both exposures.

The forest industry is one of Sweden’s most important industrial branches and the pulp- and paper mill sector constitutes an important part of this industry, which has exposure to both noise and shift work. A Norwegian study of female paper mill workers showed an increased mortality from ischemic heart disease, but did not analyze shift work or noise exposure (Langseth and Kjærheim 2006). In a Swedish study on cardiovascular risk factors, 19 female paper mill workers with a rapidly rotating shift had affected blood lipids (Axelsson et al. 2006). A previous Swedish study on pulp and paper mill employed men presented an increased risk of coronary heart disease among participants with a longer duration of shift work compared to day workers. The shift workers also had an increased risk of mortality due to stroke (Karlsson et al. 2005).

The aim of our study was to analyze mortality due to cardiovascular disease, especially acute myocardial infarction, and total mortality, in relation to exposure to shift work and noise, in a female cohort of employees in the paper mill industry.

## Methods

**Fig. 1:**
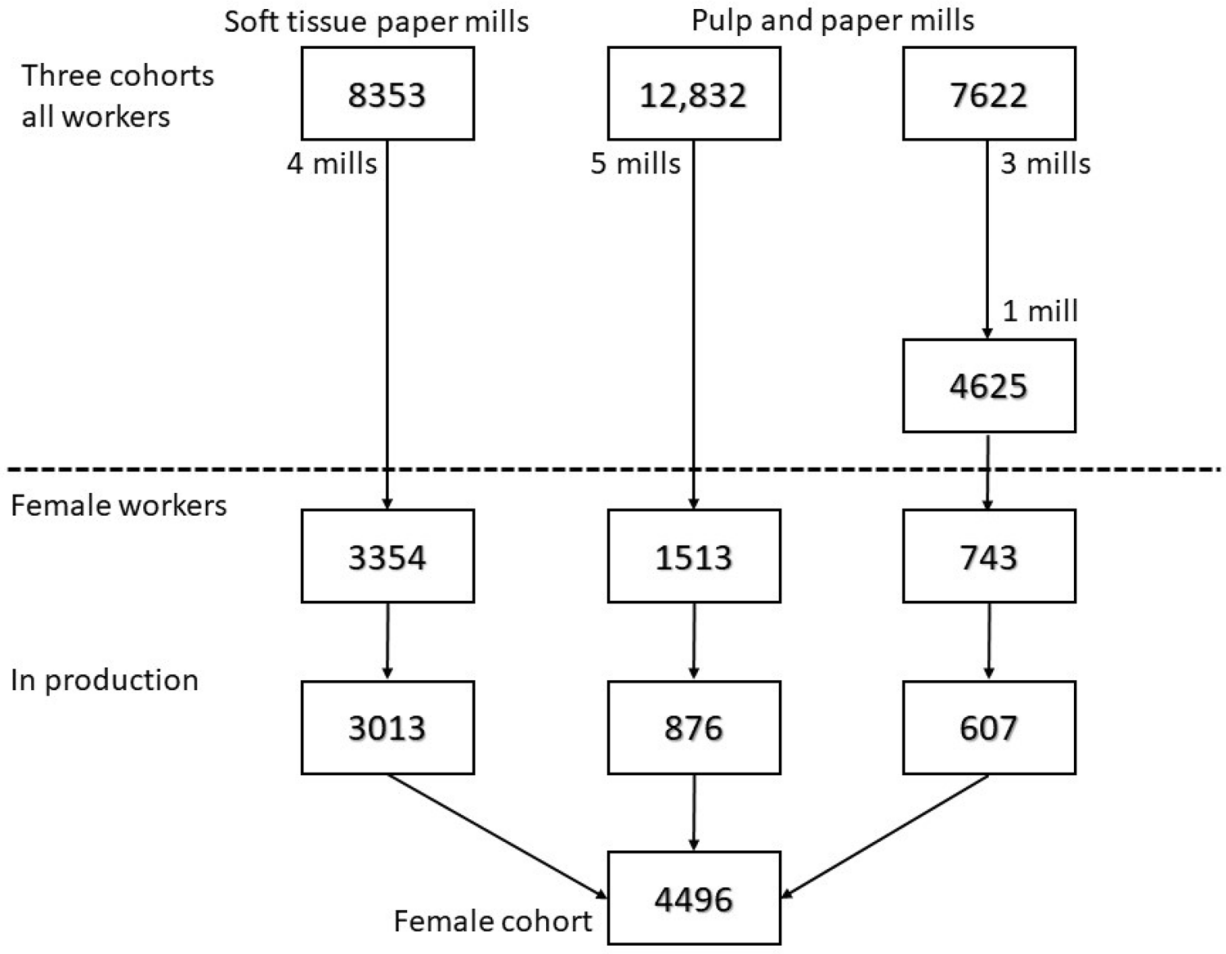
Flowchart of cohorts used for this study, number of workers, female workers and our final female cohort. Two of three pulp and paper mills in the third cohort did not include any women

We compiled a cohort of industrially employed women derived from three existing Swedish paper mill cohorts, Fig. 1. These three cohorts were previously established mainly to study airway disease (Thorén et al. 1989; Andersson et al. 2006). Historical noise exposures and shift work experience across the mills was similar. One of the three previous cohorts used in the present study was a soft tissue paper mill cohort with four mills which consisted of workers employed for more than 1 year between 1960 and 2006–2008 (different dates for different mills). Forty percent of the workers were women. The other two cohorts used were from pulp and paper mills (two sulfate and four sulfite mills), and consisted of workers employed for more than 1 year between 1950 and 1991–1999 (different dates for different mills). Thirteen percent of these workers were women. The pulp and paper mill cohorts were previously merged for mortality and cancer studies (Andersson et al. 2007, 2013).

Males were excluded, as were females who were not part of the production, i.e. office workers. Therefore, all the workers in our cohort were females, and with few exceptions, were exposed to both noise and shift work to varying degrees.

Information regarding timing and duration of employment, department and occupation was derived from personnel files. Changes in department and job title should have been noticed in the personnel files and will then be addressed by us as a new period and assessed regarding noise and shift work.

Postal questionnaires were sent to those who were alive and less than 85 years of age. At all but one pulp mill, a questionnaire was sent out in 2000 to those who were employed 1970–2000; at the soft tissue mills the questionnaire was sent out in 2006–2008 among those who were employed 1960–2006/08. The survey contained questions regarding airway symptoms and diseases, different exposures including shift work, and smoking; the soft tissue mill survey also included questions regarding noise. The response rate was 56%. In this study, the questionnaires were used mainly for shift work classification on a group level, not on an individual level, to avoid different treatment of the participants.

### Exposure assessment

#### Shift work

For each of the mills, a specific shift work job exposure matrix (JEM) was developed. The JEMs were based on personnel file data and questionnaire data on a group level for nine of the ten mills. From 53% of soft tissue mill workers and 10% of pulp mill workers, we had personnel file data with some marker for shift work, and from 29% of all we had questionnaire data of job title, department and shift work for different time periods. For one big mill, we also had detailed shift schedules for departments and sometimes job titles for most of the years. This information was compiled and used for initial versions of the JEMs. According to our own knowledge from earlier studies at the mills and from detailed historical books about all paper mills in Sweden and their production processes, we corroborated or made some changes. Then, we had focus groups at the soft tissue mills, which consisted of people responsible for the working environment, managers, and employees to discuss when certain changes were made in type of shift work and for some departments or job titles that we had no, limited or contradictory information on. For the pulp mills as those represented older times, we did not have focus groups. We contacted other researchers that also had deep knowledge of the pulp mills and discussed with them to get the best possible assessment. Women in pulp mills were often working in a few departments. The final JEM was then based on all this information, each department and period was classified as either no shift work, shift work excluding nights or shift work including nights. For the soft tissue mills with more women employed and a lot more different work tasks, 279 work locations (department and sometimes job title) were assessed. For the pulp mills, 36 work locations were assessed. The individuals were then classified first from information from personnel files, when available, and then from the JEMs. Based on this information, each workers employment period at a certain department and job title was classified as either no shift work, shift work excluding nights or shift work including nights. Up to six different periods were assessed. Every worker could then have years with all types of shift work. Production in most departments in paper mills is a continuous process that requires workers on continuous shift work, the speed and direction of shift rotation and duration of shifts have differed during times and between mills (IARC 2010). But shift work including nights means some type of continuous shift work. Shift work excluding nights means a schedule with two shifts, one early morning shift which often starts at 05.30 and one shift that starts in the afternoon and ends at 22.00. Shift work excluding nights was common among female workers until the 1980s, especially at the soft tissue mills.

#### Noise

A mill-specific noise JEM for soft tissue paper mills was developed (Neitzel et al. 2018). It was based on 100 stationary and 209 full-shift personal dosimetry measurements made at the mills by occupational hygienists from our department, Occupational and Environmental Medicine, Sahlgrenska University Hospital, as well as 812 stationary and 36 full-shift personal dosimetry measurements made by staff at the paper mills. From some mills, there were historical facility noise maps. As with the shift work JEM, information from focus groups, researchers on our own staff, and historical books was used to further improve the development of the noise JEM. Hearing protection and noise-treated control rooms were in common use from late 1980s, but the introduction of them differed in time between mills, which was taken into account in the development of the JEM. Based on these data, a semi-quantitative JEM was established in which full-shift Time-Weighted Average (TWA) noise exposures were classified into one of seven different ranked categories for each department (and sometimes occupation) and each year. The categories were: < 75 dB(A), 75–79.9 dB(A), 80–84.9 dB(A), 85–89.9 dB(A), 90–94.9 dB(A), 95–99.9 dB(A), ≥ 100 dB(A) (Neitzel et al. 2018). A noise JEM for female workers was also developed for every pulp mill where a similar procedure was done, we used information from researchers, historical books and measurements from American pulp mills and for one of the pulp mills we had detailed data on noise levels that was used. Based on information from personnel files regarding department and job title, the noise JEMs were applied to assign a TWA level for every year of mill work for every worker.

### Outcomes

Due to the Swedish unique personal identity number, linkage to the national cause of death register was possible. Personal identity numbers and exposure data of all the subjects in the cohort were sent to the National Board of Health and Welfare, which matched the cohort members with the register and sent the de-identified dataset back to us. Reference data regarding mortality for the women in the general population were also retrieved from the National Board of Health and Welfare. Outcomes studied were mortality from cardiovascular diagnoses and total mortality 1956–2013, from the Swedish Cause of Death Register. Outcomes were classified according to the diagnostic codes of the International Statistical Classification of Diseases and Related Health Problems, 6th–10th revisions (ICD-6–ICD-10). Thus, coronary heart disease was defined as ICD-6/7 codes 420 and 422.1, ICD-8/9 codes 410–414 and as ICD-10 codes I20–I25. Cerebrovascular disease, including both ischaemic stroke and intracerebral bleeding, and also subarachnoid bleeding, was defined as ICD 6/7 330–334 ICD-8/9 430–438 and ICD-10 I60–I69. From 1969 onward, acute myocardial infarction, ICD-8/9 410 and ICD-10 I21, could be analysed.

### Statistical methods

Descriptive statistics were computed as percentages within each group or median values combined with the 25th and the 75th percentile. The person-years at risk were calculated starting from first time of employment at each subject’s paper mill until time of death or the end of follow up on 31 December 2013. Person-years at risk were stratified in 5-year age groups and 1-year calendar periods. The expected numbers of deaths were calculated for these strata using all of the women in the general Swedish population as a reference. Standardized mortality ratios (SMRs) were calculated with a 95% CI with the assumption of a Poisson distribution of the observed deaths.

To allow further analysis of SMR, subjects were categorized into exposure groups based on a priori assumptions due to their shift and noise exposure. Regarding shift work, we further had to simplify the classification so that if they had had a work period with shift work including nights they were classified as that. As there were only 41 workers with only no shift work periods they were included in shift work excluding nights. Regarding noise exposure, we divided them in ever exposed more than 1 year at noise levels ≥ 90 dB(A) or not. We then divided the workers into four exposure groups: (1) shift work excluding nights, and noise levels < 90 dB(A); (2) shift work excluding nights and noise levels ≥ 90 dB(A); (3) shift work including nights and noise levels < 90 dB(A); (4) shift work including nights and noise levels ≥ 90 dB(A), Table 1. We also analyzed three levels of noise and shift work. Noise levels were grouped as: < 90 dB(A); ≥ 90 dB(A) for < 10 years; and ≥ 90 dB for ≥ 10 years, Table 1. Shift work was grouped as: shift work excluding nights; shift work including nights for < 10 years; and shift work including nights for ≥ 10 years. For additional analysis, SMR was also calculated for two different age groups, according to attained age/age at death, ≤ 65 years and > 65. The age of retirement, and thus ceased occupational exposure, has usually been 65 years in Sweden. Long-time employment, i.e. those who had been employed in the paper mill industry for more than 10 years and those who had not, was also analysed. If less than five cases, expected cases are shown in parentheses. The analyses were performed using STATA SE release 14 (Stata Statistical Software, College Station, TX, USA) and SAS 9.4 (SAS Institute, Cary, NC, USA).

**Table 1:** Exposure groups in the cohort of shift working female paper mill workers as well as the three levels of shift work and noise exposure that were analysed, number of workers and sum of person-years

Exposure group-*N* (person-years)	Shifts excluding nights	Shifts including nights		
Noise < 90 dB(A)	(1)-1316 (48,473)	(3)-681 (21,372)		1997 (69,845)
Noise ≥ 90 dB(A) < 10 years	(2)-781 (30,759)	(4)-1718 (66,658)		1897 (72,976)
Noise ≥ 90 dB(A) ≥ 10 years				602 (24,441)
		< 10 years	≥ 10 years	
	2097 (79,232)	1538 (55,508)	861 (32,523)	4496 (167,262)

## Results

The overall cohort consisted of 4496 women from the three existing paper mill cohorts. Of this total, 67% were derived from the soft tissue paper mill cohort, and the remaining 33% were derived from two pulp and paper mill cohorts (Table 2). Of the 4496 women, 64% had only worked before 1987. The total cohort median employment within the paper mill industry was 6 years, and 37% had been employed ≥ 10 years. The median TWA noise level (25–75 percentiles) was 89.2 dB(A) (84.2–92.5). The total amount of person years of follow-up was 167,262 (Table 1). Table 2 contains characteristics according to the four exposure groups; the largest exposure group were women who worked shifts including nights in noise ≥ 90 dB(A).

**Table 2:** Characteristics according to exposure groups in the cohort of shift working female paper mill workers

	(1) Shifts excl. nights, noise < 90 dB(A) *N* = 1316		(2) Shifts excl. nights, noise ≥ 90 dB(A) *N* = 781		(3) Shifts incl. nights, noise < 90 dB(A) *N* = 681		(4) Shifts incl. nights, noise ≥ 90 dB(A) *N* = 1718		Total *N* = 4496	
	%	Median (25–75 percentiles)	%	Median (25–75 percentiles)	%	Median (25–75 percentiles)	%	Median (25–75 percentiles)	%	Median (25–75 percentiles)
Pulp mills, col %	66		11		44		14		33	
Soft tissue mills, col %	34		89		56		86		67	
Age at first employment, years		23 (18–36)		30 (18–41)		23 (18–35)		22 (18–33)		23 (18–36)
Duration of employment, years		5.6 (2.4–13)		5.1 (2.4–12)		9.2 (4.1–17)		5.9 (2.5–17)		6.0 (2.6–15)
Only worked < 1987, col %	61		94		30		66		64	
Employed ≥ 10 years, col %	35		30		47		39		37	
TWA^a^ noise levels in dB(A)		82.5 (72.5–87.5)		92.5 (92.4–92.5)		87.5 (82.5–87.5)		92.5 (91.5–92.5)		89.2 (84.2–92.5)
Exposed to 85–90 dB(A), col %	38		28		69		31		38	
Years with 85–90 dB(A)^b^		3.8 (1.8–9.1)		1.1 (0.1–4.9)		7.8 (3.5–14)		7.2 (2.2–14)		na
Years with ≥ 90 dB(A)^b^		0		4.2 (2.0–9.1)		0		4.1 (2.1–9.9)		na
Shifts excl. nights, col %	97		99		12		16		54	
Years with shifts excl. nights^b^		5.3 (2.2–13)		5.0 (2.3–11)		3.7 (1.1–7.8)		3.4 (1.1–8.1)		na
Years with shifts incl. nights^b^		0		0		7.8 (3.5–15)		5.1 (2.2–14)		na

Excl. (excluding) nights means shift work without night shifts. Incl. (including) means shift work including night shifts

col %–% in each column/group

*na* not applicable

^a^TWA time weighted average

^b^Among those exposed

During the follow-up period, there were 1191 deaths (Table 3), out of which 29% were due to cardiovascular disease. For the complete female cohort, the mortality from coronary heart disease and cerebrovascular disease was not increased compared to the general population (Table 3). However, the mortality from acute myocardial infarction in the cohort was increased, SMR 1.20 (95% CI 1.01–1.41), especially for the women who died before 66 years of age, SMR 1.50 (95% CI 1.00–2.15) and among them, those employed for more than 10 years, SMR 1.83 (95% CI 1.04–2.97) (Tables 3 and 4).

**Table 3:** Mortality from cardiovascular disease and total mortality 1956–2013 among shift working female paper mill workers in different groups of shift work and noise exposure. Standardized mortality ratios (SMR) with 95% confidence intervals (CI)

TWA-time weighted average	Age at death	Coronary heart disease		Myocardial infarction (from 1969)		Cerebrovascular disease		Total mortality	
		Case	SMR (95% CI)	Case	SMR (95% CI)	Case	SMR (95% CI)	Case	SMR (95% CI)
Total cohort		228	1.05 (0.92–1.20)	144	1.20 (1.01–1.41)	116	0.95 (0.78–1.13)	1191	0.98 (0.93–1.04)
Shift and noise									
Excl. nights, TWA noise < 90 dB(A)		55	0.85 (0.64–1.11)	35	0.99 (0.69–1.37)	31	0.84 (0.57–1.20)	316	0.88 (0.78–0.98)
Excl. nights, TWA noise ≥ 90 dB(A)		74	1.08 (0.85–1.36)	49	1.31 (0.97–1.73)	33	0.87 (0.60–1.22)	360	1.05 (0.94–1.16)
Incl. nights, TWA noise < 90 dB(A)		24	1.37 (0.88–2.03)	14	1.41 (0.77–2.36)	10	0.98 (0.47–1.81)	120	1.09 (0.90–1.30)
Incl. nights, TWA noise ≥ 90 dB(A)		75	1.14 (0.90–1.43)	46	1.24 (0.91–1.66)	42	1.11 (0.80–1.50)	395	1.00 (0.90–1.10)
Attained age									
Excl. nights, TWA noise < 90 dB(A)	< 65	6	0.66 (0.24–1.44)	4	(5.5)	1	(5.4)	86	0.93 (0.74–1.15)
Excl. nights, TWA noise < 90 dB(A)	> 65	49	0.88 (0.65–1.16)	31	1.03 (0.70–1.47)	30	0.96 (0.65–1.37)	230	0.86 (0.75–0.98)
Excl. nights, TWA noise ≥ 90 dB(A)	< 65	13	1.65 (0.88–2.82)	11	2.41 (1.20–4.31)	3	(4.6)	83	1.19 (0.95–1.47)
Excl. nights, TWA noise ≥ 90 dB(A)	> 65	61	1.01 (0.77–1.29)	38	1.15 (0.82–1.58)	30	0.90 (0.60–1.28)	277	1.01 (0.90–1.14)
Incl. nights, TWA noise < 90 dB(A)	< 65	2	(3.2)	2	(2.0)	0	(1.9)	38	1.06 (0.75–1.46)
Incl. nights, TWA noise < 90 dB(A)	> 65	22	1.53 (0.96–2.32)	12	1.51 (0.78–2.64)	10	1.22 (0.58–2.24)	82	1.10 (0.88–1.37)
Incl. nights, TWA noise ≥ 90 dB(A)	< 65	17	1.49 (0.87–2.39)	12	1.66 (0.86–2.91)	7	1.00 (0.40–2.06)	129	1.02 (0.85–1.22)
Incl. nights, TWA noise ≥ 90 dB(A)	> 65	58	1.07 (0.81–1.38)	34	1.14 (0.79–1.59)	35	1.14 (0.79–1.58)	266	0.98 (0.87–1.11)
Employment > 10 years, *N* = 1671									
All exposures	< 65	21	1.41 (0.87–2.15)	16	1.83 (1.04–2.97)	4	(8.9)	124	0.89 (0.74–1.06)
All exposures	> 65	95	0.94 (0.76–1.15)	60	1.10 (0.84–1.41)	55	0.98 (0.74–1.28)	459	0.98 (0.89–1.07)

If less than five cases, expected cases are shown in parentheses. Excl. (excluding) nights means shift work without night shifts. Incl. (including) means shift work including night shifts

**Table 4:** Mortality from cardiovascular disease and total mortality 1956–2013 among shift working female paper mill workers in relation to shift work and noise

TWA-time weighted average	*N*	Coronary heart disease		Myocardial infarction (from 1969)		Cerebrovascular disease		Total mortality	
		Case	SMR (95% CI)	Case	SMR (95% CI)	Case	SMR (95% CI)	Case	SMR (95% CI)
Total cohort	4496	228	1.05 (0.92–1.20)	144	1.20 (1.01–1.41)	116	0.95 (0.78–1.13)	1191	0.98 (0.93–1.04)
Shift work									
Excluding nights	2097	129	0.97 (0.81–1.15)	84	1.15 (0.92–1.42)	64	0.86 (0.66–1.09)	676	0.96 (0.89–1.04)
Including nights < 10 years	1538	52	1.25 (0.93–1.64)	29	1.22 (0.82–1.76)	32	1.31 (0.90–1.85)	286	1.07 (0.95–1.20)
Including nights ≥ 10 years	861	47	1.13 (0.83–1.50)	31	1.33 (0.91–1.89)	20	0.85 (0.52–1.31)	229	0.96 (0.84–1.09)
Noise									
TWA Noise < 90 dB(A)	1997	79	0.96 (0.76–1.20)	49	1.08 (0.80–1.42)	41	0.87 (0.63–1.19)	436	0.93 (0.84–1.02)
TWA Noise ≥ 90 dB(A) < 10 years	1897	85	1.10 (0.88–1.36)	52	1.18 (0.88–1.55)	42	0.94 (0.68–1.27)	482	1.04 (0.95–1.13)
TWA Noise ≥ 90 dB(A) ≥ 10 years	602	64	1.13 (0.87–1.44)	43	1.41 (1.02–1.89)	33	1.06 (0.73–1.49)	273	0.99 (0.88–1.12)
Total cohort < 65 years		38	1.21 (0.85–1.65)	29	1.50 (1.00–2.15)	11	0.95 (0.58–1.04)	336	1.04 (0.93–1.15)
Shift work < 65 years									
Excluding nights		19	1.12 (0.68–1.75)	15	1.48 (0.83–2.44)	4	(10.0)	169	1.04 (0.89–1.21)
Including nights < 10 years		10	1.24 (0.59–2.28)	7	1.35 (0.54–2.79)	6	1.20 (0.44–2.60)	108	1.13 (0.93–1.37)
Including nights ≥ 10 years		9	1.38 (0.63–2.61)	7	1.72 (0.69–3.55)	1	(3.9)	59	0.89 (0.68–1.15)
Noise < 65 years									
TWA Noise < 90 dB(A)		8	0.65 (0.28–1.29)	6	0.79 (0.29–1.72)	1	(7.4)	124	0.97 (0.80–1.15)
TWA Noise ≥ 90 dB(A) < 10 years		19	1.48 (0.89–2.32)	15	1.82 (1.02–3.00)	7	0.92 (0.16–2.22)	166	1.20 (1.02–1.40)
TWA Noise ≥ 90 dB(A) ≥ 10 years		11	1.70 (0.85–3.04)	8	2.26 (0.97–4.45)	3	(3.9)	46	0.80 (0.59–1.07)

Standardized mortality ratios (SMR) with 95% confidence intervals (CI). If less than five cases, expected cases are shown in parentheses

Female workers exposed to shift work excluding nights and noise exposure < 90 dB(A) did not have an increased mortality from acute myocardial infarction (Table 3). Among those who were exposed to noise ≥ 90 dB(A) for more than 10 years, the mortality from myocardial infarction was SMR 1.41 (95% CI 1.02–1.89) (Table 4). For those who were below 66 years at death and exposed to noise ≥ 90 dB(A), the mortality from myocardial infarction was even more pronounced, with an SMR of 1.95 (95% CI 1.24–2.93), data not shown. When participants who had worked shifts including nights more than 10 years were analysed, the SMR from myocardial infarction was 1.33 (95% CI 0.91–1.89) (Table 4). For the combined exposure of noise ≥ 90 dB(A) and shift work excluding nights the SMR from myocardial infarction was 1.31 (95% CI 0.97–1.73) and among them, for the participants below 66 years of age, the mortality from myocardial infarction was SMR 2.41 (95% CI 1.20–4.31) (Table 3). This exposure group mainly worked before 1987 (Table 2).

The total mortality was increased among participants who died before 66 years and exposed to noise ≥ 90 dB(A) for < 10 years, SMR 1.20 (95% CI 1.02–1.40). Total mortality in the cohort was not increased.

## Discussion

This cohort study among female paper mill workers, who have been exposed to noise and shift work, showed a significantly increased mortality from acute myocardial infarction, but not from other cardiovascular diseases. This finding was most pronounced among those exposed to high levels of noise with a long duration of employment and who died before 66 years of age, which suggests an association with work. Shift workers without night shifts and noise exposure less than 90 dB(A) did not have an increased mortality.

This study confirms earlier findings of an increased mortality from ischemic heart disease in the paper mill industry. A Norwegian study of female paper mill workers showed an increased mortality from ischemic heart disease SMR 1.22, 95% CI 1.03–1.43) (Langseth and Kjærheim 2006). In a Swedish pulp mill study, male workers had an increased mortality from acute myocardial infarction (Andersson et al. 2007) but the female workers had no significantly increased risk when followed until 2001. Most of these females are included in the present study but now followed until 2013. However, noise and shift work were not assessed in these two studies.

There was no significantly increased mortality in cerebrovascular disease, in agreement with a commentary summarizing the conflicting evidence on whether occupational exposure to noise increases the risk of stroke (Kolstad et al. 2013). A meta-analysis on shift work and cardiovascular disease showed a slightly increased risk of ischaemic stroke RR 1.05 (95% CI 1.01–1.09 but concluded there were no studies on hemorrhagic stroke and shift work (Vyas et al. 2012).

Our study is one of very few that has considered the simultaneous exposure to both shift work and noise among industrially employed women in a large cohort with long time of follow-up. It is rarely acknowledged in shift work studies that industrial work is frequently accompanied by exposure to noise. A Finnish study of industrially employed men showed an increased risk of ischemic heart disease when exposure to shift work occurred as well as when exposure for noise occurred, and when both exposures occurred together (Virkkunen et al. 2006). Almost all subjects in our study were exposed to shift work excluding or including nights, but there were relatively few subjects that were exposed to shift work only and not to noise, all the participants were exposed to some degree of noise. Hence, it was difficult to separate the effects of shift work from the effects of noise. Joint exposure to shift work and noise is likely common in other industrial settings as well and should be considered in studies. A synergistic effect between occupational noise and shift work is also possible (Attarchi et al 2012).

The women with a combined exposure of noise ≥ 90 dB(A) and shift work excluding nights had an increased SMR from myocardial infarction, especially the women below 66 years of age. Shift work excluding nights includes morning shifts starting at 05.30 in our study, which could imply disturbed sleep (Kecklund and Axelsson 2016). Also, this exposure group mainly worked before 1987. Hearing protection use and noise-treated control rooms were rare before the 1980s. The gradually increased use of hearing protection and the introduction of control rooms has reduced the noise exposure over time in the paper mills (Neitzel et al. 2018). In a large sawmill cohort study, where exposure to noise was investigated, increased mortality from acute myocardial infarction was found, with a stronger association in a subgroup working before hearing protection came into use (Davies et al. 2005). The risk increased with years worked and with noise level [> 85 dB(A), > 90 dB(A), > 95 dB(A)], and the highest mortality was observed during employment at the saw mills. The authors also found that smoking did not appear to confound the associations. They did not report if there was any shift work.

Noise exposure levels were assigned based on the results of the JEM, which was based partly on previously performed noise measurements and likely contains less bias than JEMs based on self-reports or expert judgement. There remains the possibility for misclassification of the exposure estimates, which could attenuate our risk estimates. No individual-level data on hearing protection use were applied, since it was available only from part of one mill, which was a weakness of the study. Correct use of hearing protection devices can substantially reduce noise exposures, and our inability to account for use of such devices may have biased our risk estimates down.

Another weakness of our study, and of other shift work studies (Sun et al. 2018), was the difficulty of classifying shift work in detail. It was not possible to identify each person’s individual schedules over the study period, nor was it possible to study different types of rotation of shift work. Personal survey data regarding shift work were not used to avoid different treatment of the subjects; as with the hearing protector data, there was personal survey data on shift work only from a limited portion of the cohort. Furthermore, the lack of complete data on smoking was a weakness of the study, and prevented us from controlling for this confounder in the whole cohort. However, earlier subgroup analyses on 1686 subjects from the studied cohort, where it was possible to adjust for smoking from survey data, did not substantially change risk estimates, compared to unadjusted for smoking, but had low power (data not shown).

We analyzed only women in this study, which limits the external validity and reduces the power of the study. However, many shift work studies have been performed on women working as nurses but rarely regarding women working in industries. Furthermore, previous research on noise exposure in industrial settings has been performed mainly on men. We used the women in the general population as a reference when calculating SMRs. This could pose a risk of bias due to a healthy worker effect. The general population includes women who do not work due to illness, our studied cohort only include workers, this could bias, decrease, our risk estimates.

It is important with customized personal protective equipment and with enclosure of noise as far as possible to keep the noise exposure low in industrial settings as well as other workplaces. Also, shift work schedules should be optimized and medical controls should be performed on shift workers. In Sweden, employers nowadays are obliged to offer medical controls to workers who work during the night.

## Conclusions

Our results show an increased mortality from myocardial infarction among women working in the production of paper mills, who had been exposed to noise and shift work, compared to women in the general population. Especially for participants in the study who died before 66 years of age and who were exposed to full-shift noise levels of 90 dB(A) or more. There was no significantly increased mortality in cerebrovascular disease.

This study gives further evidence and strengthens the association between occupational noise, shift work and coronary heart disease. It is important to reduce exposure to noise and optimize shift schedules.
